# Blood Glutamate Scavenging With Pyruvate as a Novel Preventative and Therapeutic Approach for Depressive-Like Behavior Following Traumatic Brain Injury in a Rat Model

**DOI:** 10.3389/fnins.2022.832478

**Published:** 2022-02-14

**Authors:** Dmitry Frank, Benjamin F. Gruenbaum, Ilan Shelef, Vladislav Zvenigorodsky, Olena Severynovska, Ron Gal, Michael Dubilet, Alexander Zlotnik, Ora Kofman, Matthew Boyko

**Affiliations:** ^1^Department of Anesthesiology and Critical Care, Soroka University Medical Center, Ben-Gurion University of the Negev, Be’er Sheva, Israel; ^2^Department of Anesthesiology and Perioperative Medicine, Mayo Clinic, Jacksonville, FL, United States; ^3^Department of Radiology, Soroka University Medical Center, Ben-Gurion University of the Negev, Be’er Sheva, Israel; ^4^Department of Physiology, Faculty of Biology, Ecology and Medicine, Dnepropetrovsk State University, Dnepropetrovsk, Ukraine; ^5^ Department of Psychology, Zlotowski Center for Neuroscience, Ben-Gurion University of the Negev, Be’er Sheva, Israel

**Keywords:** depression, glutamate scavenging, neuroprotection, pyruvate, traumatic brain injury

## Abstract

Depression is a common and serious complication following traumatic brain injury (TBI). Both depression and TBI have independently been associated with pathologically elevated extracellular brain glutamate levels. In the setting of TBI, blood glutamate scavenging with pyruvate has been widely shown as an effective method to provide neuroprotection by reducing blood glutamate and subsequent brain glutamate levels. Here we evaluate pyruvate as a novel approach in the treatment and prevention of post-TBI depression-like behavior in a rat model. Rats were divided into five groups: (1) sham-operated control with pyruvate, (2) sham-operated control with placebo, (3) post-TBI with placebo, (4) post-TBI given preventative pyruvate, and (5) post-TBI treated with pyruvate. These groups had an equal number of females and males. Rats were assessed for depressive-like behavior, neurological status, and glutamate levels in the blood and brain. Post-TBI neurological deficits with concurrent elevations in glutamate levels were demonstrated, with peak glutamate levels 24 h after TBI. Following TBI, the administration of either prophylactic or therapeutic pyruvate led to reduced glutamate levels, improved neurologic recovery, and improved depressive-like behavior. Glutamate scavenging with pyruvate may be an effective prophylactic and therapeutic option for post-TBI depression by reducing associated elevations in brain glutamate levels.

## Introduction

The majority of survivors of moderate and severe traumatic brain injury (TBI) suffer from chronic neuropsychiatric consequences, including cognitive defects, depression, anxiety, social withdrawal and aggression (Tateno et al., 2003; McAllister, 2008; Jorge and Arciniegas, 2014; Hicks et al., 2019; Rauen et al., 2020). While these behavioral sequelae may at first be attributable to the emotional burdens of physical disability, these symptoms are not correlated with the severity of the initial injury or with pain (Bodnar et al., 2019) and can persist for decades (Hoofien et al., 2001; Koponen et al., 2002). Despite their significant impacts on functional recovery, quality of life, and resumption of employment (Rivara et al., 2011), these chronic neuropsychiatric conditions following TBI are often overlooked, undiagnosed and untreated.

Depressive disorders are generally treated by targeting the serotoninergic, adrenergic, and/or dopaminergic systems with medication that increases synaptic access of these neurotransmitters (Robinson et al., 1984; Currier et al., 1992; Andersen et al., 1994; Wiart et al., 2000). However, treatment for depressive disorders is effective in approximately two thirds of patients. For those suffering from depression following TBI, the selective serotonin reuptake inhibitor Sertraline (Zoloft) was found to be no more effective than placebo (Fann et al., 2017). As post-TBI depression remains difficult to manage, novel therapeutic approaches that specifically target this and related neuropsychiatric conditions have been of great clinical interest.

A growing body of evidence points to the involvement of the glutamatergic system in the etiology and treatment of TBI and depression, both independently and in parallel (O’Neil et al., 2018). Glutamate levels in the brain have been shown to contribute to the pathophysiology and neurological dysfunction seen after TBI (Zauner et al., 1996b; Koura et al., 1998; Zhang et al., 2001; Shutter et al., 2004; Mao et al., 2019). Post-TBI excess extracellular glutamate release leads to cell swelling, apoptosis, and neuronal death (Zauner et al., 1996a; Koura et al., 1998), and the maintenance of glutamate homeostasis is critical in improving neurological outcome (Zauner et al., 1996b; Hong et al., 2001; Zhang et al., 2001; Shutter et al., 2004; Mao et al., 2019). Depression and many mood disorders are similarly affected by the glutamatergic system (Levine et al., 2000; Krystal et al., 2002; Sanacora et al., 2003, 2012; Mitani et al., 2006; Maeng and Zarate, 2007; Pittenger et al., 2007; Mitchell and Baker, 2010; Zarate et al., 2010; Machado-Vieira et al., 2012; McCarthy et al., 2012; Tokita et al., 2012) of evidence indicates that future therapeutic options for depression will be comprised of modalities based on this system (Sanacora et al., 2008; Gruenbaum et al., 2020). Recent literature suggests that a susceptibility to depression may be caused by glutamatergic disturbances after TBI (O’Neil et al., 2018). Therefore, limiting excess glutamate concentrations following TBI may be a vital strategy to target both the neurologic and psychiatric progression of the condition.

Neurological motor symptoms of TBI have been shown to be attenuated by decreasing glutamate levels or function in the brain with dextorphan (Faden et al., 1989), *N*-methyl-D-aspartate (NMDA) antagonists (Mei et al., 2018), stimulation of excitatory amino acid transporters (EAATs) (Goodrich et al., 2013), or antibiotics and other drugs that block calcium channels or glutamate release (McConeghy et al., 2012; Hicks et al., 2019). However, these treatments can also limit the essential effects of glutamate, leading to adverse side effects (Ikonomidou and Turski, 2002; Hardingham and Bading, 2003; Muir, 2006). For example, human clinical trials of NMDA receptor antagonists have not only failed to demonstrate clinical neuroprotective efficacy but led to worsened neurological outcome and an increased mortality rate following TBI (Morris et al., 1998; Muir, 2006). Moreover, other preclinical studies have found that direct or indirect stimulation of NMDA receptors mitigated the severity of neurological deficits in hippocampal-based memory in adult rats (Temple and Hamm, 1996; Biegon et al., 2004) and in rat pups (Sta Maria et al., 2017; Biegon et al., 2018) after TBI.

An alternative approach is to eliminate excess toxic glutamate, rather than interfering with ongoing excitatory transmission *via* receptor antagonists. This can be accomplished by enhancing the brain-to-blood glutamate efflux, which occurs naturally *via* the endothelial transport systems, to eliminate excess glutamate from the brain’s interstitial fluid (Teichberg, 2007; Teichberg et al., 2009). Glutamate co-substrates pyruvate and oxaloacetate convert glutamate into its inactive form 2-ketoglutarate *via* blood resident enzymes glutamate-pyruvate transaminase and glutamate-oxaloacetate transaminase (Gonzalez et al., 2005; Leibowitz et al., 2012; Gray et al., 2014). Previous studies have established an association between brain glutamate and blood glutamate levels (Shaw et al., 1995; Ferrarese et al., 2001). A reduction in blood glutamate helps to form an ideal glutamate concentration gradient that causes excess glutamate to move from the brain’s extracellular fluid into the blood (Zlotnik et al., 2011a,2012a; Rogachev et al., 2012; Boyko et al., 2014). This process impedes secondary brain injury that can occur as a result of glutamate neurotoxicity (O’Kane et al., 1999; Teichberg et al., 2009; Boyko et al., 2014).

Glutamate reduction, unlike the use of NMDA receptor antagonists, does not impact glutamate receptors or glutamate-mediated synaptic activity. Instead, this process only removes pathologically-elevated glutamate levels in the brain without impeding the function of neural circuits that depend on glutamate transmission (Leibowitz et al., 2012; Boyko et al., 2014; Zhumadilov et al., 2015). Known as blood glutamate scavenging, this method for reduction of excess glutamate has been proposed as an effective method to ameliorate neurological conditions after TBI (Zlotnik et al., 2007, 2008, 2009, 2010, 2012b) and depressive symptoms after stroke (Frank et al., 2019a; Gruenbaum et al., 2020). The aim of this study was to employ a novel approach of blood glutamate scavenging with pyruvate for the prevention and treatment of post-TBI depressive-like behaviors in a rat model. We further analyzed the impact of gender differences on the development of post-TBI depressive-like behaviors and on subsequent treatment with blood glutamate scavenging.

## Materials and Methods

### Animals

The experiments were conducted in accordance with the recommendation of the Declarations of Helsinki and Tokyo and the Guidelines for the Use of Experimental Animals of the European Community. The experiments were approved by the Animal Care Committee of Ben-Gurion University of the Negev (Beer-Sheva, Israel). A total of 134 male and 133 female Sprague-Dawley rats were used in this experiment. All rats weighed between 300 and 350 g. Purina Chow and water were made available *ad libitum*. The temperature in the room was maintained at 22°C, with a 12 h light–dark cycle. All the tests were conducted in the dark phase between 8 am and 4 pm.

### Experimental Design

**FIGURE 1:**
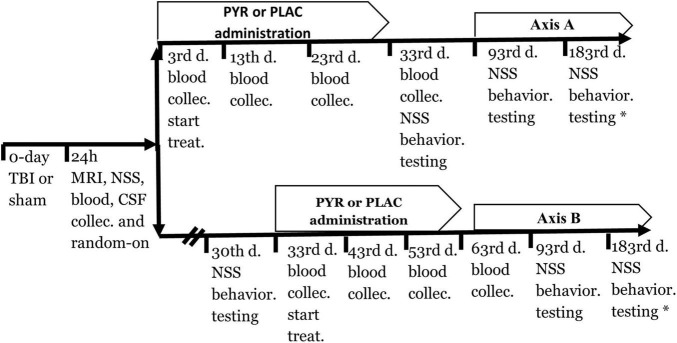
A timeline of the protocol for preventative (axis A) and treatment (axis B) approach. CSF, Cerebrospinal fluid; NSS, Neurological severity score; PLAC, Placebo; PYR, Pyruvate; TBI, Traumatic brain injury.

The timeline of the experiment is illustrated in Figure 1. All rats were divided into two main groups, sham-operated and TBI. The rats were randomly assigned, but each group had an equal number of females and males (Table 1). 24 h after induction of TBI or sham surgery, all rats were divided into five groups: (1) sham-operated control group given pyruvate, (2) sham-operated control group given placebo, (3) post-TBI control group given placebo, (4) post-TBI group given preventative pyruvate, (5) post-TBI group treated with pyruvate (Table 1). Each of the five groups was randomly divided into two subgroups: (A) a group for behavioral tests and (B) a group for testing blood and cerebrospinal fluid (CSF), and outcomes from magnetic resonance imaging (MRI) with anesthesia (Table 1). At 24 h after TBI or sham protocol, we collected a sample of CSF and blood from the rats in subgroup B. On day 3 of the study, two groups (the post-TBI group given preventative pyruvate and the sham-operated control group given pyruvate) began to receive pyruvate for 30 days (Figure 1, Axis A). Within subgroup A, behavioral tests were performed after the completion of treatment at 1-month post-TBI, and 2 months after the completion of treatment. After the TBI induction or sham operation, the rats from the therapeutic protocol received no treatment for a month. After 1-month, behavioral tests (subgroup A) or blood CSF measurements (subgroup B) were taken, followed by treatment with pyruvate (Figure 1, Axis B) at a dose described below. Behavioral tests at 6 months were performed only for the sham-operated control group given placebo and post-TBI rats given placebo (Figure 1).

**TABLE 1:** The total number of rats in each of the experimental groups.

Study groups	Experimental procedures		Number of rats	
	MRI, CSF, and blood collection	Neuro-behavioral tests	Female	Male
Sham-operated controls given pyruvate	10f	15f	25	25
	10m	15m		
Sham-operated controls given placebo	10f	15f	25	25
	10m	15m		
Post-TBI rats given placebo	10f	15f	25	25
	10m	15m		
Post-TBI rats given preventative pyruvate	10f	15f	25	25
	10m	15m		
Post-TBI rats treated with pyruvate	10f	15f	25	25
	10m	15m		
The total number of rats			125	125

### Drugs and Doses

Pyruvate (Sigma Israel Chemicals, Rehovot, Israel, catalog number P2256) was kept at a temperature of 2–4°C prior to use. Immediately before administration, it was dissolved in drinking water. Doses of 180 mg/kg/day were administered to rats in the experimental groups divided into two daily doses of 90 mg/kg for 30 days. A fresh solution of pyruvate was made every 12 h. The placebo groups received an equal dose of water without pyruvate. The dose of pyruvate was based on previous data that demonstrated by magnetic resonance spectroscopy that a dose of 180 mg/kg/day was optimal for reducing blood and brain glutamate by about 25–35% (Frank et al., 2019a).

### Traumatic Brain Injury

Traumatic brain injury was performed, as previously described (Jones et al., 2008; Kabadi et al., 2010; Frank et al., 2021a,b). Rats received inhaled isoflurane as anesthetic with 5% for induction and 1.5–2.5% for maintenance, with equal parts medical air and oxygen. Prior to incision, the scalp was infiltrated with 0.5% bupivacaine. It was then perforated and reflected laterally with the left temporal muscle, while the underlying periosteum was dissected to reveal the skull. Craniotomy was performed at 5-mm using a trephine (Roboz Surgical Instrument Co., Gaithersburg, MD, United States) fastened to the drill bit of an electrical drill (Stoelting, Wood Dale, IL, United States). The center of the craniotomy was positioned 4 mm lateral and 4 mm posterior to bregma. A Luer 3-way stopcock was fixed and additionally held in place by cyanoacrylate adhesive and dental acrylic. The injury was then effected by a pressure pulse of 2.2 atmospheres (Jones et al., 2008; Kabadi et al., 2010). TBI was induced by a fluid-percussion device over 21–23 ms through the 3-way stopcock. The fluid pulse from the piston plunger, through involvement by the pendulum, was enacted *via* continuous saline fluid into the dura to allow for efficient transmission of the pressure pulse. Rats in the sham-operated control groups underwent the same procedure but without the administration of the fluid pulse.

Rats were monitored by a pulse-oximeter during the surgery to ensure uninterrupted measurements of heart rate and blood oxygen levels. After TBI induction, the incision was sutured, and the rats were allowed to recover from anesthesia.

### Neurological Severity Score

Two blinded observers calculated Neurological Severity Score (NSS), as previously described (Boyko et al., 2011a,2013a; Ohayon et al., 2012; Zlotnik et al., 2012a; Frank et al., 2021b). Points were assigned for motor function and behavioral changes for an overall score between 0, indicating an intact neurological state, and 25, representing highest neurological impairment. The following criteria were evaluated: the ability to exit a circle (3-point scale), gait on a wide surface (3-point scale), gait on a narrow surface (4-point scale), effort to remain on a narrow surface (2-point scale), reflexes (5-point scale), seeking behavior (2-point scale), beam walking (3-point scale), and beam balance (3-point scale).

### Sucrose Preference Test

The sucrose preference test was performed as described previously as a method to evaluate anhedonia, which reflects depressive-like symptoms, in a rodent model (Boyko et al., 2013a,^2015^). Two bottles of sucrose solution were placed in each rat’s cage, consisting of 1% (w/v) solution. The rat became acclimated to having two bottles in the cage, which allowed the rat to avoid neophobia during the sucrose preference test, for which two bottles were necessary. Similarly, one of the bottles was replaced by water for 24 h so that the rat could adjust to having one bottle of water and one bottle of sucrose. After this habituation, the rats were deprived of food and water for 12 h. At 9:00 am, the sucrose preference test was performed. The rats were housed in individual cages with free access to two bottles, one with 100 ml of sucrose solution (1% w/v) and the other with 100 ml of water, for 4 h. After this period, the volume (ml) of the consumed sucrose solution and water was recorded. Sucrose preference was calculated as sucrose preference (%) = sucrose consumption (ml)/[sucrose consumption (ml)+water consumption (ml)] × 100% (Boyko et al., 2013b,2019b).

### Open Field Test

The standard open field test evaluates locomotor, exploratory, and anxiety-related depressive-like behaviors in animal models based on novel conditions (Boyko et al., 2013a). The open field test measures exploratory activity in a novel environment. The open field boxes were round black plastic arenas 2 m in diameter, 60 cm high walls situated in a darkened room. For analysis, the arena was cleaned with 10% ethanol after each behavioral recording.

A video camera was mounted 200 cm above the open field arena and recorded all experiments. Locomotor activity was recorded for 5 min by a Logitech HD Pro Webcam C920. Analysis after the recording was performed with Ethovision XT software (Noldus, Wageningen, Netherlands) (Frank et al., 2019b). The recordings were analyzed based on total distance traveled.

### Magnetic Resonance Imaging

Diffusion-weighted imaging and T2 MRI were performed at 48 h following TBI, as described previously (Frank et al., 2019a). The rats underwent general anesthesia and were maintained with 1.5% isoflurane in oxygen. A 3T MRI was used (Ingenia, Philips Medical Systems, Best, Netherlands) using an eight-channel receive-only coil. Localizing T2w turbo spin echo (TSE) sequences were obtained in sagittal and coronal planes with TR/TE = 3,000/80 ms, turbo factor = 15, water-fat shift = 1.6 pixels, resolution (freq × phase × slice) = 0.47 mm × 0.41 mm × 2.0 mm and one average for a scan time of 1:00 min. In the axial direction the scan parameters included repetition time/echo time (TR/TE) = 3,000/80 ms, turbo factor = 14, water-fat shift = 1.6 pixels, resolution (freq × phase × slice) = 0.37 mm × 0.33 mm × 2.0 mm. Four averages were acquired for a scan time of 4:54 min. Diffusion tensor imaging in 6 directions was performed in the axial direction using a multi-shot STimulated Echo Acquisition Mode (STEAM) spin-echo, echo-planar sequence with repetition time/mixing time/echo time (TR/TM/TE) = 1,355/15.0/143 ms, SENSitivity Encoding (SENSE) reduction factor = 1.5, turbo factor = 19, b = 1,000 s/mm2, resolution (freq × phase × slice) = 0.55 mm × 0.55 mm × 2.0 mm with spectrally-selective fat suppression. Five signal averages were acquired for a scan time of 8:40 min. T2 perfusion studies were obtained using a dynamic, single-shot gradient-echo epi sequence with spectrally-selective fat suppression. The scan parameters were TR/TE = 1,300/40 ms, resolution (freq × phase × slice) = 0.64 mm × 0.69 mm × 2.0 mm, and one signal average giving a scan time of 1.3sec/dynamic. A total of 150 dynamics were acquired for a scan time of 3:19 min. We utilized the Intellispace Portal workstation (V5.0.0.20030, Philips Medical Systems, Best, Netherlands) for the post-processing of the perfusion studies.

### Magnetic Resonance Imaging Analysis

An expert blinded to the groups performed image analysis. We generated quantitative apparent diffusion coefficient (ADC) maps, in units of square millimeters per second, in Philips software package (Ingenia, Philips Medical Systems, Best, Netherlands). Analysis was performed using ImageJ software (version 1.50i, National Institutes of Health, Bethesda, Maryland), as previously described (Boyko et al., 2019c). These thresholds indicated all pixels of ADC characteristics on each slice. The viability thresholds were 0.53X10-3mm^2^/s for ADC images (Bardutzky et al., 2005; Boyko et al., 2019c). Calculation of lesion volume was performed by the RICH method and included the correction for tissue swelling, according to the following formula (Boyko et al., 2013b):


$$C\,o\,r\,r\,e\,c\,t\,e\,d\,l\,e\,s\,i\,o\,n\,v\,o\,l\,u\,m\,e=\frac{L\,e\,s\,i\,o\,n\,v\,o\,l\,u\,m\,e\times C\,o\,n\,t\,r\,a\,l\,a\,t\,e\,r\,a\,l\,h\,e\,m\,i\,s\,p\,h\,e\,r\,e\,s\,i\,z\,e}{I\,p\,s\,i\,l\,a\,t\,e\,r\,a\,l\,h\,e\,m\,i\,s\,p\,h\,e\,r\,e\,s\,i\,z\,e}$$

Calculation of brain edema was also performed by the RICH method. The calculation of brain edema by the RICH technique was done by comparing the contralateral and ipsilateral hemispheres, and performed using the following formula (Boyko et al., 2011a):


$$B\,r\,a\,i\,n\,e\,d\,e\,m\,a=\frac{V\,o\,l\,u\,m\,e\,o\,f\,t\,h\,e\,r\,i\,g\,h\,t\,h\,e\,m\,i\,s\,p\,h\,e\,r\,e-V\,o\,l\,u\,m\,e\,o\,f\,t\,h\,e\,l\,e\,f\,t\,h\,e\,m\,i\,s\,p\,h\,e\,r\,e}{V\,o\,l\,u\,m\,e\,o\,f\,t\,h\,e\,l\,e\,f\,t\,h\,e\,m\,i\,s\,p\,h\,e\,r\,e}$$

The lesion volume and brain edema were measured as a percentage of the total brain (Boyko et al., 2019a).

### Determination of Blood Glutamate

Whole blood (200 μl aliquot) had its protein removed by adding an equal volume of ice-cold 1 M perchloric acid, followed by utilization of a centrifuge at 10,000 × *g* for 10 min at 4°C. The supernatant was obtained for future analysis if necessary, and adjusted to pH 7.2, with 2 M K2CO3, and stored at −80°C.

To measure the glutamate concentration, the fluorometric method of Graham and Aprison (1966) was used (Graham and Aprison, 1966). A 60 μl aliquot from the perchloric acid supernatant was combined with 90 μl of a 0.3 M glycine; 0.25 M hydrazine hydrate buffer adjusted to pH 8.6 with 1 M H2SO4 and containing 11.25 U of glutamate dehydrogenase in 10 mM Nicotinamide adenine dinucleotide. After incubation for 30 to 45 min at room temperature, the fluorescence was measured at 460 nm with excitation at 350 nm. A glutamate standard curve was established with concentrations ranging from 0 to 6 μM. All determinations were done at least in duplicates (Boyko et al., 2011b).

### Blood Sample Collection

Blood was collected from the tail vein for the determination of blood glutamate levels *via* a 24- guage Neoflon (Becton Dickinson, Helsingborg, Sweden) catheter. After the blood sample was collected, the catheter was removed from the vein (Boyko et al., 2012).

### Cerebrospinal Fluid Sample Collection

Rats were anesthetized and the cisterna magna was cannulated, as previously described (Boyko et al., 2012), and 0.1 to 0.2 ml of CSF were gently aspirated.

### Determination of Cerebrospinal Fluid Glutamate

Fresh CSF (110 μl) was mixed with perchloric acid (25 μl) of 0.3 M, and then centrifuged at 10,000 × *g* for 10 min at 4°C. The pellet was discarded and the supernatant was collected, adjusted to pH 7.2 with 12.5 μl of 2 M K2CO3 and stored at −80°C for later analysis (Boyko et al., 2012). Analysis was performed by fluorometric method as described above for blood samples.

### Statistical Analysis

Statistical analysis was performed with the SPSS 20 package (SPSS Inc., Chicago, IL, United States). The Kolmogorov–Smirnov test was used, to consider the number of rats in each group for deciding the appropriate test for the comparisons between the different parameters. For non-parametric data, we used the transformation test or other suitable tests. The significance of comparisons between groups were determined using the Kruskal–Wallis and Mann–Whitney (for nonparametric data) and one-way ANOVA with Bonferroni *post hoc* test or the Student’s *t*-tests (for parametric data). Mortality rate was analyzed with chi-square and Fisher’s exact tests. Results were considered statistically significant when *P* < 0.05, and highly significant when *P* < 0.01.

## Results

### Mortality

The survival rate was calculated in the first 3 days following TBI or sham-operated procedure. During this period, the rats were not administered pyruvate. The mortality rate in sham-operated control rats was 0% in both gender groups, which was significantly lower than male (10.71%, *p* = 2.6E-02, chi-square and Fisher’s exact test, 2-sided) and female (9.64%, *p* = 2.5E-02, chi-square and Fisher’s exact test, 2-sided) rats following TBI.

### Neurological Severity Score

**FIGURE 2:**
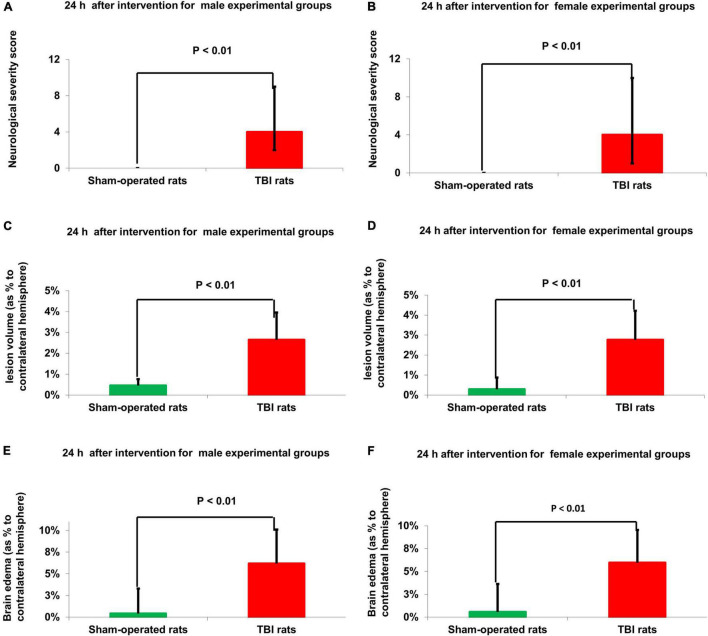
Neurological outcome **(A,B)** and MRI-determined lesion volume **(C,D)** and brain edema **(E,F)**. Compared to sham-operated controls, the NSS at 24 h was significantly greater in male [*p* < 0.01 **(A)**] and female [*p* < 0.01 **(B)**] rats after TBI. The data are measured as a count and expressed as median and 25–75 percentile range. Compared to sham-operated rats, the lesion volume at 24 h was significantly greater in the male [*p* < 0.01 **(C)**] and female [*p* < 0.01 **(D)**] TBI groups. The data is expressed as a mean percentage of the contralateral hemisphere ± SD. Compared to sham-operated rats, the brain edema at 24 h was significantly greater in the male [*p* < 0.01, **(E)**] and female [*p* < 0.01 **(F)**] TBI groups. The data are expressed as a mean percentage of the contralateral hemisphere ± SD. TBI: Traumatic brain injury.

There were no baseline neurological deficits observed in any of the rats before TBI or sham-operated procedure. The sham-operated control groups did not show any neurological deficit at any time point throughout the experiment. Compared to sham-operated controls, the NSS at 24 h was significantly greater in male [4(2–5) *n* = 75 vs. 0(0-0) *n* = 50, *U* = 45, *p* = 2.7E-21, *r* = 0.85] and female [4(3–6) *n* = 75 vs. 0(0-0) *n* = 50, *U* = 0, *p* = 4.5E-22, *r* = 0.86] rats after TBI, according to Mann–Whitney test (Figures 2A,B). No statistically significant differences were found between the 15 male and female groups, at time points of 30, 90, and 180 days, according to Kruskal–Wallis one-way analysis (see Figure 1 and Table 1). The data are measured as a count and expressed as median and 25–75 percentile range.

### Magnetic Resonance Imaging-Determined Lesion Volume

Compared to sham-operated rats, the lesion volume at 24 h was significantly greater in the female [2.71% ± 1.29% vs.0.46% ± 0.24%, *t*(48) = −7.21, *p* = 3.4E-09] and male [2.68% ± 1.32% vs. 0.45% ± 0.28%, *t*(48) = −7.26, *p* = 3E-09] TBI groups, according to Student’s *t*-test (Figures 2C,D). The data are expressed as a mean percentage of the contralateral hemisphere ± SD.

### Magnetic Resonance Imaging-Determined Brain Edema

Compared to sham-operated rats, the brain edema at 24 h was significantly greater in the female [5.98% ± 2.83% vs. 0.57% ± 0.29%, *t*(48) = −5.5, *p* = 1.4E-06] and male [6.15% ± 3.26% vs. 0.41% ± 0.24%, *t*(48) = −5.6, *p* = 1E-06] TBI groups, according to Student’s *t*-test (Figures 2E,F). The data are expressed as a mean percentage of the contralateral hemisphere ± SD.

### Concentration of Cerebrospinal Fluid Glutamate

**FIGURE 3:**
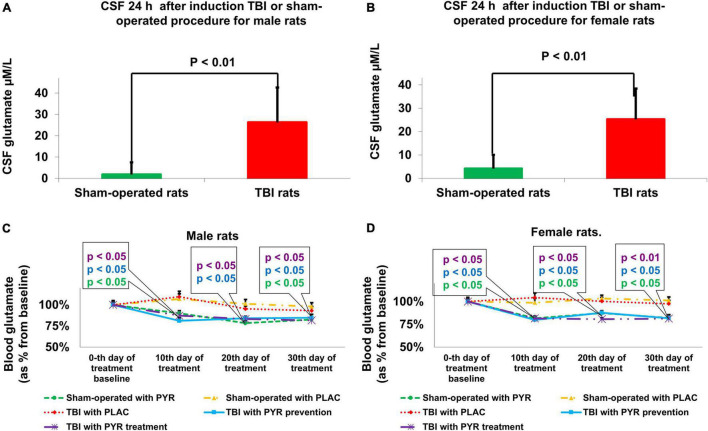
Brain **(A,B)** and blood **(C,D)** glutamate concentrations. Compared to sham-operated rats, the concentrations of CSF glutamate at 24 h was significantly greater in the male [*p* < 0.01, **(A)**] and female [*p* < 0.01 **(B)**] TBI groups. The data are measured in μM/L and expressed as mean ± SD. Blood glutamate levels were decreased in male [*p* < 0.05 **(C)**] and female [*p* < 0.05 **(D)**] groups that received preventative or therapeutic pyruvate. The data are measured in μM/L presented as a percentage from baseline and expressed as mean ± SEM. CSF, Cerebrospinal fluid; PLAC, Placebo; PYR, Pyruvate; TBI, Traumatic brain injury.

Compared to sham-operated rats, the concentration of CSF glutamate at 24 h was significantly greater in the female [25.27 μM/L ± 13.13 μM/L vs. 3.6 μM/L ± 6.28 μM/L, *t*(48) = −6.9, *p* = 1.2E-08] and male [26.27 μM/L ± 16.39 μM/L vs. 2.1 μM/L ± 5.76 μM/L, *t*(48) = −6.4, *p* = 6.1E-08] TBI groups, according to Student’s *t*-test (Figures 3A,B). The data are measured in μM/L and expressed as mean ± SD.

### Concentration of Blood Glutamate

At baseline, there were no significant differences in blood glutamate concentration between treatment groups.

Compared to sham-operated rats, the concentration of blood glutamate at 24 h was significantly greater in the female [121% ± 20% vs. 100% ± 18%, *t*(48) = 3.87, *p* < 0.01] and male [114% ± 18% vs. 100% ± 17%, *t*(48) = 2.75, *p* < 0.01] TBI groups, according to Student’s *t*-test. The data are measured in μM/L presented as a percentage from sham-operated rats and expressed as mean ± SD.

For male rats, at day 10 after pyruvate administration or placebo protocol, there were significant differences in blood glutamate levels between sham-operated rats given placebo (107% ± 8.6%), sham-operated rats given pyruvate (80.4% ± 4.4%), post-TBI given placebo (109.5% ± 9.4%), post-TBI rats given preventative pyruvate (77.6% ± 4.5%), and post-TBI rats treated with pyruvate (80.3% ± 6.1%) [Kruskal–Wallis, χ^2^ (4) = 12.9, *p* = 1.2E-02]. A subsequent Mann–Whitney test indicated that male blood glutamate levels were significantly decreased in the sham-operated rats given pyruvate (*U* = 21, *p* = 2.8E-02, *r* = –0.49), post-TBI rats given preventative pyruvate (*U* = 18, *p* = 1.6E-02, *r* = −0.02), and post-TBI rats treated with pyruvate (*U* = 22, *p* = 3.4E-02, *r* = −0.47), compared to sham-operated rats given placebo. At day 20, there were significant differences in male blood glutamate levels between sham-operated rats given placebo (101.2% ± 7.3%), sham-operated rats given pyruvate (82.7% ± 5.1%), post-TBI given placebo (105.2% ± 4.8%), post-TBI rats given preventative pyruvate (80.6% ± 3.5%) and post-TBI rats treated with pyruvate (77.4% ± 3.5%) [Kruskal–Wallis, χ2 (4) = 11, *p* = 2.6E-02]. A subsequent Mann–Whitney test indicated that male blood glutamate levels were significantly decreased in the post-TBI rats given preventative pyruvate (*U* = 24, *p* = 4.9E-02, *r* = −0.44) and post-TBI rats treated with pyruvate (*U* = 20, *p* = 2.3E-02, *r* = −0.51), compared to the sham-operated control group given placebo. Also on day 20, male blood glutamate levels in the sham-operated rats given pyruvate were lower than in the sham-operated control rats given placebo, although this difference did not reach statistical significance. At day 30, there were significant differences in male blood glutamate levels between sham-operated rats given placebo (99.4% ± 5.9%), sham-operated rats given pyruvate (75.9% ± 5.2%), post-TBI given placebo (93.1% ± 7.1%), post-TBI rats given preventative pyruvate (77.9% ± 5.4%) and post-TBI rats treated with pyruvate (79.9% ± 3.6%) [Kruskal–Wallis, χ2 (4) = 10.1, *p* = 3.9E-02]. A subsequent Mann–Whitney test indicated that male blood glutamate levels were significantly decreased in the sham-operated controls given pyruvate (*U* = 16, *p* = 1E-02, *r* = −0.58), post-TBI rats given preventative pyruvate (*U* = 75, *p* = 2.3E-02, *r* = −0.51) and post-TBI rats treated with pyruvate (*U* = 75, *p* = 2.3E-02, *r* = −0.51), compared to sham-operated rats given placebo (Figure 3C).

For female rats, at day 10, there were significant differences in blood glutamate levels between sham-operated rats given placebo (98.7% ± 6.7%), sham-operated rats given pyruvate (80% ± 4.3%), post-TBI given placebo (104.5% ± 7%), post-TBI rats given preventative pyruvate (80.1% ± 3%) and post-TBI rats treated with pyruvate (78.7% ± 4.4%) [Kruskal–Wallis, χ2 (4) = 12.8, *p* = 1.2E-02]. A subsequent Mann–Whitney test indicated that at day 10, female blood glutamate levels were significantly decreased in the sham-operated controls given pyruvate (*U* = 23, *p* = 4.1E-02, *r* = −0.46), post-TBI rats given preventative pyruvate (*U* = 23, *p* = 4.1E-02, *r* = −0.46), and post-TBI rats treated with pyruvate (*U* = 24, *p* = 4.9E-02, *r* = −0.45), compared to sham-operated rats given placebo. At day 20, there were significant differences in female blood glutamate levels between sham-operated rats given placebo (103.4% ± 5.1%), sham-operated rats given pyruvate (81.1% ± 5.7%), post-TBI given placebo (100.3% ± 6.1%), post-TBI rats given preventative pyruvate (86.4% ± 4.9%) and post-TBI rats treated with pyruvate (83.2% ± 5%) [Kruskal–Wallis, χ2 (4) = 11.8, *p* = 1.9E-02]. A subsequent Mann–Whitney test indicated that at day 20, female blood glutamate levels were significantly decreased in the sham-operated controls given pyruvate (*U* = 18, *p* = 1.6E-02, *r* = −0.54), post-TBI rats given preventative pyruvate (*U* = 24, *p* = 4.9E-02, *r* = −0.44), and post-TBI rats treated with pyruvate (*U* = 18, *p* = 1.6E-02, *r* = −0.54), compared to sham-operated rats given placebo. At day 30, there were significant differences in female blood glutamate levels between sham-operated rats given placebo 101.2% ± 5.4%), sham-operated rats given pyruvate (82.9% ± 4%), post-TBI given placebo (97.6% ± 5.3%), post-TBI rats given preventative pyruvate (80.4% ± 5.6%) and post-TBI rats treated with pyruvate (74.2% ± 3.7%) [Kruskal–Wallis, χ2 (4) = 14.6, *p* = 5.7E-03]. A subsequent Mann–Whitney test indicated that at day 30, female blood glutamate levels were significantly decreased in the sham-operated controls given pyruvate (*U* = 19, *p* = 1.9E-02, *r* = −0.52), post-TBI rats given preventative pyruvate (*U* = 21, *p* = 2.8E-02, *r* = −0.49) and post-TBI rats treated with pyruvate (*U* = 11 *p* = 3.2E-02, *r* = −0.66), compared to sham-operated controls given placebo (Figure 3D).

As expected (Puig et al., 2000), blood glutamate levels in post-TBI rats treated with placebo were not statistically significantly different than in the sham-operated control rats treated with placebo. The data are measured in μfvM/L presented as a percentage from baseline and expressed as mean ± SEM.

### Sucrose Preference

**FIGURE 4:**
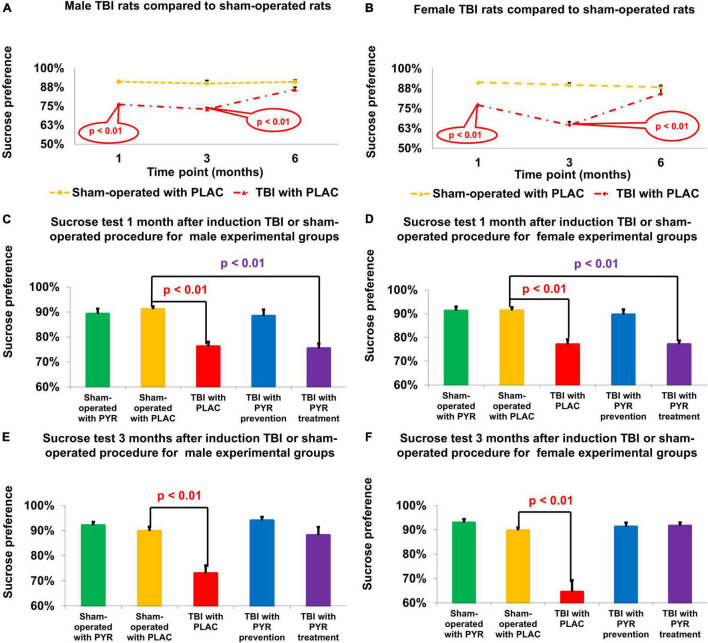
Sucrose preference test. There was a decrease in sucrose preference at 1 and 3 months following TBI for male **(A)** and female **(B)** rats given placebo compared to sham-operated controls (*p* < 0.01). For male **(C)** and female **(D)** rats at day 30, there was a significant decrease in sucrose preference in post-TBI rats given placebo (*p* < 0.01) and post-TBI rats treated with pyruvate (*p* < 0.01) compared to sham-operated controls given placebo. For male **(E)** and female **(F)** rats at day 90, there was a significant decrease in sucrose preference in post-TBI rats given placebo (*p* < 0.01) compared to sham-operated controls given placebo. The data are measured as a percentage and presented as mean ± SEM. PLAC, Placebo; PYR, Pyruvate; TBI, Traumatic brain injury.

For male rats at day 30, a one-way ANOVA showed a significant difference in the percentage of sucrose preference between the study groups *F*(4,65) = 13.5, *p* = 4.8E-08. *Post hoc* analysis with a Bonferroni test showed a significant decrease between post-TBI rats given placebo (76.2% ± 1.9%, *p* = 4E-05) and post-TBI rats treated with pyruvate (75.4% ± 2%, *p* = 8.7E-06) compared to sham-operated controls given placebo (91.1% ± 1.2%). At day 90, a one-way ANOVA showed a significant difference in the percentage of sucrose preference between the study groups *F*(4,65) = 11.11, *p* = 6.3E-07. *Post hoc* analysis with a Bonferroni test showed a significant decrease in post-TBI rats given placebo (73% ± 3.1%, *p* = 6.5E-05) compared to sham-operated controls given placebo (89.9% ± 1.7%) (Figures 4A,C,E).

For female rats at day 30, a one-way ANOVA showed a significant difference in the percentage of sucrose preference between the study groups *F*(4, 68) = 16.27, *p* = 2.2E-09. *Post hoc* analysis with a Bonferroni test showed a significant decrease in post-TBI rats given placebo (77% ± 2.2%, *p* = 7.5E-06) and post-TBI rats treated with pyruvate (77.1% ± 1.6%, *p* = 8.2E-06), compared to sham-operated controls given placebo (91.4% ± 1.3%). At day 90, a one-way ANOVA showed a significant difference in the percentage of sucrose preference between the study groups *F*(4,65) = 21.29, *p* = 3.0E-11. *Post hoc* analysis with a Bonferroni test showed a significant decrease in post-TBI rats given placebo (64.5% ± 4.8%, *p* = 1.4E-08) compared to sham-operated controls given placebo (89.8% ± 1.2%) (Figures 4B,D,F). The data are measured in ml presented as percentage and expressed as mean ± SEM.

### Open-Field Test

**FIGURE 5:**
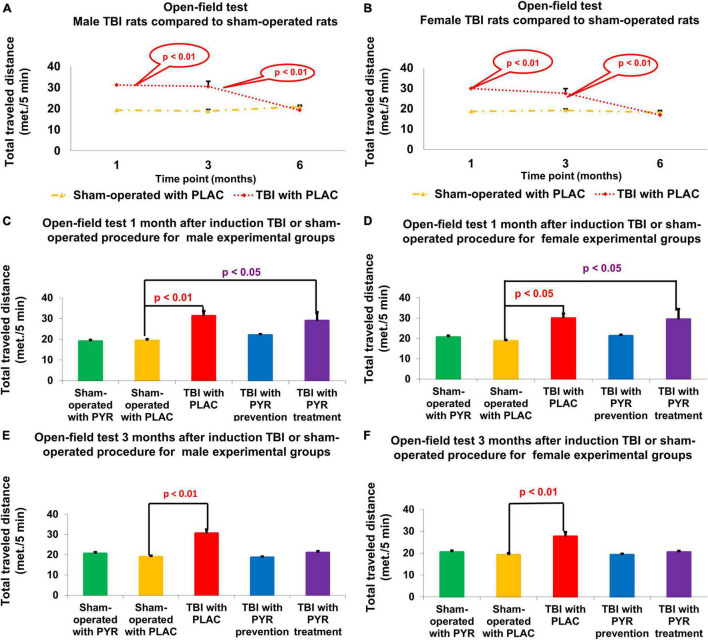
Open-field test. There was an increase in the total traveled distance at 1 and 3 months following TBI for male **(A)** and female **(B)** rats given placebo compared to sham-operated controls (*p* < 0.05). For male **(C)** and female **(D)** rats at day 30, there was a significant increase in total distance traveled in post-TBI rats given placebo (*p* < 0.01) and post-TBI rats treated with pyruvate (*p* < 0.01) compared to sham-operated controls given placebo. For male **(E)** and female **(F)** rats at day 90, there was a significant increase in total distance traveled in post-TBI rats given placebo (*p* < 0.01) compared to sham-operated controls given placebo. The data are measured as cm/5 min and presented as mean ± SEM. PLAC, Placebo; PYR, Pyruvate; TBI, Traumatic brain injury.

For male rats at day 30, a one-way ANOVA showed a significant difference in the total distance traveled between the study groups *F*(4,72) = 6.49, *p* = 1.6E-08. *Post hoc* analysis with a Bonferroni test showed a significant increase in post-TBI rats given placebo (31.21 m ± 2.5 m, *p* = 2.9E-03) and post-TBI rats treated with pyruvate (28.93 m ± 4.25 m, *p* = 2.9E-02), compared to sham-operated controls given placebo (19.3 m ± 0.77 m). At day 90, a one-way ANOVA showed a significant difference in the total distance traveled between the study groups *F*(4,70) = 19.74, *p* = 6.5E-11. *Post hoc* analysis with a Bonferroni test showed a significant increase in post-TBI rats given placebo (30.52 m ± 1.94 m, *p* = 8.1E-10) compared to sham-operated controls given placebo (18.84 m ± 0.72 m) (Figures 5A,C,E).

For female rats at day 30, a one-way ANOVA showed a significant difference in the total distance traveled between the study groups *F*(4,72) = 4.4, *p* = 3.1E-03. *Post hoc* analysis with a Bonferroni test showed a significant increase in post-TBI rats given placebo (29.89 m ± 2.33 m, *p* = 2.3E-02) and post-TBI rats treated with pyruvate (29.37 m ± 5.12 m, *p* = 3.5E-02), compared to sham-operated controls given placebo (18.65 m ± 0.69 m). At day 90, a one-way ANOVA showed significant difference in the total distance traveled between the study groups *F*(4,69) = 9.25, *p* = 4.8E-06. *Post hoc* analysis with a Bonferroni test showed a significant increase in distance traveled by post-TBI rats given placebo (27.55 m ± 2.06 m, *p* = 1.6E-05) compared to sham-operated controls given placebo (19.19 m ± 0.88 m) (Figures 5B,D,F). The data are measured as meter/5 min and presented as mean ± SEM.

## Discussion

In this study, we investigated blood glutamate scavenging activity from pyruvate administration and its mechanisms as a viable option for antidepressant treatment in a rat model of post-traumatic depression. Specifically, we studied the effects of pyruvate on anhedonia and elevated locomotor activity (Bhatt et al., 2017) as a consequence of post-traumatic behavioral mood disorders. Additionally, we considered brain glutamate levels and blood glutamate scavenging, MRI findings, and neurological outcomes between male and female rodent groups, and we used both a prophylactic and a therapeutic pyruvate treatment protocol. Our results determined that pyruvate likely has an antidepressant effect on the brain *via* its participation in blood glutamate scavenging.

Our hypotheses on the following neurological conditions were confirmed by our study. We determined that the mortality rate in the TBI group was higher than in the sham group. Cerebral edema and lesion volume were significantly higher in the TBI group compared to the sham group (Figures 2C–F). We observed that neurological deficits were significantly greater in the TBI group compared to the sham group, which spontaneously recovered by 1 month (Figures 2A,B and section results > neurological performance). In addition, the concentration of glutamate was increased in the cerebrospinal fluid at 24 h as a consequence of TBI (Figures 3A,B). We have previously shown that the administration of pyruvate is effective in reducing cerebrospinal fluid glutamate levels in rodent models of subarachnoid hemorrhage (Boyko et al., 2012) and stroke (Frank et al., 2019a; Gruenbaum et al., 2020). A significant process of blood glutamate scavenging occurred in groups that received pyruvate treatment compared to placebo groups (Figures 3C–D). All the above results applied to both the male and female cohorts (Figures 2, 3). At days 10–30 following TBI, the glutamate levels in rats given placebo did not differ from the levels in naïve rats treated with placebo. In previous studies in the setting of stroke, an increase in blood glutamate levels was seen in the first 24 h, but the levels dropped to baseline at 48 h and beyond (Puig et al., 2000).

To study the efficacy of pyruvate as an antidepressant therapy, we used two behavioral tests. The first was the sucrose preference test that measures the level of anhedonia, one of the most common symptoms of depressive disorders (American Psychiatric Pub, 2013). The sucrose preference test is a standard test for assessing anhedonia in rat models and has allowed for the development of new therapeutic antidepressant treatments (Gururajan et al., 2019). In our study, the TBI rats developed anhedonia at higher rates compared to sham rats and then spontaneously recovered 6 months after TBI (Figures 4A,B). In contrast, the post-TBI rats who were administered pyruvate prophylactically showed no symptoms of anhedonia and did not differ from the results of the sham rat group at 1 and 3 months following TBI (Figures 4B–F).

Traumatic brain injury rats that received pyruvate only after they developed anhedonia symptoms, showed a therapeutic effect of the treatment at 3 months. Thus, pyruvate has proven to be effective in two approaches: in the prophylactic treatment protocol as well as the therapy protocol (Figure 4). Pyruvate showed equal rates of efficacy in both the male and female cohorts (Figure 4).

The second behavioral test which we used was an open field test, a common method used to detect high emotionality and locomotor hyperactivity (Ramamoorthy et al., 2008) in post-TBI rats (Lewen et al., 1999; Pandey et al., 2009) and mice (Li et al., 2006; Pullela et al., 2006; Tucker et al., 2016). These manifestations are associated with depressive status (Pandey et al., 2009) and show high response rates to antidepressant drugs (Lewen et al., 1999; Pandey et al., 2009; Bhatt et al., 2017; Jindal et al., 2017). The hyperlocomotion that we recorded in the TBI group during the open field test is attributable to damage caused by the brain insult to the cerebral cortex, striatum, and olfactory bulbs (Viggiano, 2008). An increase in total distance traveled in the olfactory bulbectomized model of depression is well documented in the literature (Kalueff and Tuohimaa, 2004). Assessment of hyperlocomotive behavior as a consequence of TBI is also used to verify new models of TBI (Kane et al., 2012).

Our study supports the hypothesis that hyperlocomotion after TBI is associated with dysregulation of the glutamatergic system, in particular by high levels of extracellular glutamate. The association between dysregulation of the glutamatergic system and hyperlocomotion has been widely reported (Takahata and Moghaddam, 2003; Abekawa et al., 2007; Hackler et al., 2010; Egerton et al., 2020). It was previously observed that activation of metabotropic glutamate receptors increases both horizontal and vertical locomotor activity and this activity is impeded by administration of a receptor antagonist, fluphenazine (Kim and Vezina, 1997). Gainetdinov et al. (2001) showed that drugs that enhance glutamatergic transmission, such as positive modulators of L-α-amino-3-hydroxy-5-methylisoxazole-4-propionate glutamate receptors, suppress the hyperactivity of mice lacking the dopamine transporter (Gainetdinov et al., 2001). The involvement of the glutamate system in the development of attention-deficit disorder, hyperactivity, and other behavioral motor disorders has also been previously described (Procaccini et al., 2013; Maksimovic et al., 2014a,b; Miller, 2019; Aitta-Aho et al., 2019). Halberstadt et al. (2011) demonstrated that loss of mGlu5 receptor activity either pharmacologically or through gene deletion leads to locomotor hyperactivity in mice. These studies strongly indicate that dysregulation of the glutamatergic system, alone or in combination with other major neurotransmitter systems such as dopamine, GABA, and the serotonin system, may induce hyper-locomotive effects that are controlled by drugs that regulate glutamate homeostasis (Tucker et al., 2016). Although the precise brain circuitry and pharmacological targets involved in the suppression of locomotor behavior require further elucidation, our data support the possibility that glutamatergic transmission in the hippocampus could be therapeutically applied to dampen the hyper-excitable hippocampus and other brain circuitries.

In our study, we found that TBI rats were more likely to travel farther distances in the open field compared to the sham group until 6 months following TBI. In addition, more TBI rats developed hyperlocomotion activity compared to sham rats and then spontaneously recovered 6 months after TBI. In contrast, the rats after TBI from the protocol of preventive treatment with pyruvate showed no symptoms of hyperlocomotive activity and did not differ from sham rats at 1 and 3 months following TBI. TBI rats that were in the treatment group and did not receive pyruvate after TBI developed symptoms of hyperlocomotion and only then began to receive pyruvate as a therapeutic approach. Thus, pyruvate showed its efficacy both as a prophylactic protocol and as a therapeutic protocol. Pyruvate showed equal effective results in both male and female cohorts.

The similarities between the male and female cohorts in the outcomes of the sucrose preference test and the open field test elucidate our understanding of gender differences concerning depression and anxiety. Women tend to suffer more often from major depressive disorder (Kovacs et al., 1989; Weissman et al., 1993; Bebbington, 1998; Merikangas et al., 2010) and anxiety (Angst and Dobler-Mikola, 1985; Kessler et al., 1994; Bruce et al., 2005). In rodent models, different rat strains can display significant gender disparities in models of depression (Kokras and Dalla, 2014), though it is generally observed that female rodents appear more active in the open field test, with less anxiety (Ter Horst et al., 2009; Kokras and Dalla, 2014). In our study, the use of the sucrose preference test in addition to the open field test assisted in developing more comprehensive neurological findings.

While it was outside of the scope of this study, we have previously observed that women display lower levels of blood glutamate concentration at baseline, and in conditions such as amyotrophic lateral sclerosis, rheumatoid arthritis, and growth hormone deficiency (Stover and Kempski, 2005). We have also determined that progesterone and estrogen have neuroprotective properties that act to reduce blood glutamate levels (Zlotnik et al., 2011b; Tsesis et al., 2013). We followed recommendations in the literature to include both sexes in this model (Rubin and Lipton, 2019), and, therefore our results accurately show the possible regulatory effects of pyruvate in similar ways across both groups. We hypothesize that more research on the topic of gender differences will support the use of pyruvate as a pharmacological approach that addresses depression for both men and women.

In our study, we began treatment on the third day after TBI. Usually, however, new therapeutic modalities are administered in the first hours after a brain injury (Tucker et al., 2016). We based our methodology on previous evidence that pyruvate has a neuroprotective effect in models of stroke and subarachnoid hemorrhage and, when administered in the first hours, reduces cerebral edema, infarction zone and blood brain barrier breakdown (Frank et al., 2019a). A reduction in damage to the brain tissue after pyruvate administration can potentially affect the development of behavioral outcomes after TBI. To neutralize the effect of histological outcomes on behavioral ones, we started pyruvate administration on the third day after TBI.

In summary, we have provided significant evidence that the process of blood glutamate scavenging by pyruvate induces antidepressant properties. These properties result in the prevention or treatment of anhedonia and hyperlocomotion that are caused by glutamate deregulation after TBI in rats. These conditions are symptoms of depressive-like conditions in rodent models. When analyzed in conjunction with previously observed neuroprotective properties of blood glutamate scavenging, it has become more apparent that blood glutamate scavengers should be considered as a viable treatment option for post-TBI depression.

## Data Availability Statement

The raw data supporting the conclusion of this article will be made available by the corresponding author (MB), upon reasonable request.

## Ethics Statement

The animal study was reviewed and approved by the experiments were approved by the Animal Care Committee of Ben-Gurion University of the Negev (Be’er Sheva, Israel).

## Author Contributions

DF, BG, OK, and MB: study conception, data collection, data analysis, manuscript writing and editing, and final approval of manuscript. IS, VZ, OS, RG, MD, and AZ: data collection, data analysis, manuscript editing, and final approval of manuscript. All authors contributed to the article and approved the submitted version.

## Conflict of Interest

The authors declare that the research was conducted in the absence of any commercial or financial relationships that could be construed as a potential conflict of interest.

## Publisher’s Note

All claims expressed in this article are solely those of the authors and do not necessarily represent those of their affiliated organizations, or those of the publisher, the editors and the reviewers. Any product that may be evaluated in this article, or claim that may be made by its manufacturer, is not guaranteed or endorsed by the publisher.

## Abbreviations

ADC: apparent diffusion coefficient
CSF: cerebrospinal fluid
EAATs: excitatory amino acid transporters
MRI: magnetic resonance imaging
NSS: Neurological severity score
NMDA: *N*-methyl-D-aspartate
TR/TE: repetition time/echo time
TR/TM/TE: repetition time/mixing time/echo time
SENSE: SENSitivity Encoding
STEAM: STimulated Echo Acquisition Mode
TBI: traumatic brain injury
TSE: turbo spin echo.
